# HbA1c-based personalization of glucose management in critical illness: a target trial emulation

**DOI:** 10.62675/2965-2774.20260001

**Published:** 2026-08-31

**Authors:** Joseph Furler, Frank M Gao, Aniket Nadkarni, Meg Stevens, Dinesh Pandey, Hossein Jahanabadi, Ary Serpa

**Affiliations:** 1 Austin Hospital Department of Intensive Care Melbourne Australia Department of Intensive Care, Austin Hospital - Melbourne, Australia.; 2 Austin Hospital Department of Endocrinology Melbourne Australia Department of Endocrinology, Austin Hospital - Melbourne, Australia.; 3 Austin Hospital Data Analytics Research and Evaluation Centre Melbourne Australia Data Analytics Research and Evaluation Centre, Austin Hospital - Melbourne, Australia.

**Keywords:** Intensive care unit, Glucose management, Target trial emulation, Critical care

## Abstract

**Objective:**

To evaluate the effect of HbA1c-guided glycaemic control on 30-day intensive care unit mortality in intensive care unit patients.

**Design:**

Target trial emulation using the parametric g-formula. Setting: A single tertiary academic hospital intensive care unit in Melbourne, Australia. Patients: All adults admitted between January 2019 and December 2023 with an admission HbA1c measurement and complete intensive care unit admission/discharge data. Interventions: Three glucose control strategies were compared: natural course (usual care), NICE-SUGAR target (8 - 10mmol/L), and personalised HbA1c-guided target (usual glycaemia ± 0.8mmol/L).

**Results:**

Among 3,541 patients (64% male, median age 68 years), mortality was 6.46% (95%CI 4.16% - 8.83%) under the natural course, 6.52% (95%CI 4.40% - 8.82%) with NICE-SUGAR, and 6.56% (95%CI 4.40% - 8.85%) with HbA1c-guided targets. Absolute risk differences *versus* usual care was 0.10% (95%CI -0.26% - 0.93) for HbA1c-guided strategy. Subgroup analyses by diabetes status, admission type, illness severity, and septic shock showed no survival benefit.

**Conclusion:**

In this large single-centre target trial emulation, personalising glycaemic targets based on admission HbA1c did not improve 30-day intensive care unit mortality compared with either usual care or NICE-SUGAR targets. These findings suggest that HbA1c-guided glucose control is unlikely to confer meaningful survival benefit under current implementation approaches.

## INTRODUCTION

Hyperglycaemia is common in critically ill patients and is associated with worse outcomes, including infection, impaired wound healing, and mortality.^(1)^ While early trials suggested benefit from intensive glycaemic control, subsequent large multicentre trials, including Normoglycemia in Intensive Care Evaluation-Survival Using Glucose Algorithm Regulation (NICE-SUGAR), showed harm or no benefit, particularly due to increased hypoglycaemia.^(2)^ As a result, current guidelines recommend a conventional target of < 10mmol/L (180mg/dL),^(3)^ but despite its well-established pathophysiology, the optimal approach to glycaemic control in the intensive care unit (ICU) remains controversial, particularly regarding whether treatment targets should be universal or personalized.^(4)^

A universal glycaemic target may not suit all patients. Observational data suggest that the optimal glucose level may differ based on chronic glycaemic status, with patients with diabetes tolerating higher glucose levels.^(5)^ Hemoglobin A1c (HbA1c), a measure of long-term glycaemic exposure, reflects mean glucose levels over approximately 3 months and is a key biomarker in diabetes diagnosis and prognosis.^(6)^ Its availability at hospital or ICU admission offers a potential opportunity to personalise glycaemic targets according to a patient's usual glycaemia, rather than applying a fixed threshold to all.

The CONTROLING trial tested this hypothesis by randomizing ICU patients to either conventional control (< 10mmol/L [180mg/dL]) or personalized control based on HbA1c.^(7)^ Although the trial found no overall mortality benefit, evidence from selected ICU populations suggests potential benefit of personalizing glycaemic targets.^(8-10)^ In cardiac surgical and neurosurgical cohorts, tighter or individualized control has been associated with improved outcomes.^(8-10)^ Moreover, observational data demonstrate complex interactions between chronic and acute glycaemia.^(11)^ For instance, the glycaemic level associated with lowest mortality appears to be higher in patients with diabetes than in those without, implying that a universal glucose target might inadvertently overtreat some patients while undertreating others.^(12)^ These findings raise the question of whether specific ICU sub-populations might still benefit from a personalized approach, a hypothesis that can now be evaluated through robust target trial emulations using real-world data.

The causal research question addressed by this target trial emulation is: among critically ill adult patients admitted to the ICU, what is the effect of maintaining glucose trajectories within personalized HbA1c-based targets compared with conventional glucose control strategies, including NICE-SUGAR targets and observed clinical practice, on ICU mortality within 30 days of ICU admission? Accordingly, the primary objective of this study was to evaluate the effect of HbA1c-guided glycaemic control on 30-day intensive care unit mortality in intensive care unit patients.

## METHODS

### Study design

We conducted a target trial emulation assessing the effect of personalized glycaemic control based on HbA1c, on clinical outcomes of critically ill patients. Details of the trial emulation protocol can be found in table 1. A target trial emulation was chosen in place of a traditional observational study to reduce the effect of selection/immortal time bias and allow stronger causal inferences to be made.^(13)^ By analyzing retrospective data via a target trial emulation design we are also able to utilize existing records to provide faster insight compared to a standard clinical trial. This study was approved by the Austin Hospital Human Research Ethics Committee (VicTRI-16351-HREC/111269/Austin-2024), with a waiver of informed consent.

**Table 1:** Summary of the protocol of the target trial assessing the impact of different glycaemic control strategies on mortality

Components	Hypothetical randomized trial	Emulation
Eligibility	All adult patients admitted to the ICUs of the Austin Hospital, Melbourne between January 2019 and December 2023. Patients were excluded only if their data in ICU admission or discharge date and time was missing	Same as hypothetical trial
Treatment strategies	Conventional management of hyperglycaemia (usual care) − HbA1c strategy (maintenance at usual glycaemia ± 0.8mmol/L) NICE-SUGAR strategy (maintenance at 8 -10mmol/L)	Same as hypothetical trial. In a sensitivity analysis, different definitions of usual glycaemia were investigated
Treatment assignment	Patients are randomly assigned to one of the strategies. Stratification was performed based on clinical characteristics. The treatment team was aware of the assigned strategy	We assumed that patients were randomly assigned within levels of the different baseline variables
Follow-up	The follow-up started at the time of assignment to a glycaemic control strategy and ended at either at − Death − Discharge from ICU alive − 30 days of admission, whichever comes first	Follow-up starts at the first time when all eligibility criteria are met
Primary outcomes	30-day ICU mortality	Same as hypothetical trial
Causal contrast	Per protocol effect	Observational analogue of the per protocol effect
Statistical analysis	Intention-to-treat analysis. In the per-protocol analysis, patients were censored when they deviated from their assigned strategy. The per-protocol effect was estimated after adjustment for baseline variables and for time-dependent variables associated with adherence to a strategy maintaining glycemia at a personalized level.	The per-protocol effect was estimated under full adherence using the parametric g-formula. The value of each density function for all possible covariate histories was estimated under parametric modelling assumptions. The approximated sum over all histories was subsequently calculated via Monte Carlo simulation

ICU - intensive care unit; HbA1c - Hemoglobin A1c; NICE-SUGAR - Normoglycemia in Intensive Care Evaluation-Survival Using Glucose Algorithm Regulation.

### Patients

All adult patients (≥ 18 years old) admitted to the ICU of the Austin Hospital, Melbourne, were eligible for inclusion. If a patient had multiple admissions, only the first admission was considered for inclusion. In addition, only patients with an HbA1c measure at hospital admission were included in the analysis. At our institution, HbA1c testing is supported by an institutional protocol embedded in the electronic medical record (EMR). Since approximately mid-2013, HbA1c has been routinely measured in hospitalized patients aged ≥ 54 years if no HbA1c result was available within the preceding 3 months. This automated testing protocol facilitates systematic assessment of chronic glycaemic status and reduces reliance on clinician-directed testing alone. Patients were excluded only if their data in ICU admission or discharge date and time was missing.

### Data collection

All patient baseline and outcome data were drawn from the Australian and New Zealand Intensive Care Society (ANZIC) Adult ICU Patient Database (APD) and Austin EMR. Data collected included patient demographics, anthropometric measurements, admission category, comorbidities including diabetic status, organ support, and clinical outcomes. Insulin dosage and administration, and glucose measurements were drawn from the EMR. All available measurements (from pathology, blood gas and capillary) were retrieved. All data was timestamped. The HbA1c at hospital admission was used to calculate the usual glycaemia for each patient using the formula below:


$$Usual\;glycaemia\left(\frac{mmol}{L}\right)=\frac{\left(28.7\times \text{HbA}1\text{c}-46.7\right)}{18}$$

### Intervention

In the HbA1c strategy group, the glycaemia target was set to usual glycaemia (calculated based on the formula above) ± 0.8mmol/L.^(7)^ In the usual care group (natural course), the actual glycaemia achieved during ICU stay was considered. Finally, in the NICE-SUGAR strategy, the glycaemia target was set to 8 to 10mmol/L.^(2)^

### Outcomes and study endpoints

The primary outcome of interest was 30-day ICU mortality. Discharge from ICU was considered a competing event because it precluded the later occurrence of the primary outcome. Patients were followed from the ICU admission (Day Zero) until either death, ICU discharge, or Day 30 in the ICU, whichever occurred first. In all analyses, time zero was the ICU admission and the maximum follow-up time was 30 days from ICU admission. The 30-day period was considered because more than 99% of the patients had less than 30 days of length of stay. The follow-up was divided in blocks of 6 hours.

### Analysis plan

Maintenance of glycaemia within proposed targets is a time-dependent exposure. In addition, there are time-dependent confounders affected by prior treatment that also predict future glucose levels and future outcome, conditional on past treatment. The use of standard regression methods in this situation results in biased estimates. Target trial emulation using parametric g-formula is an accepted statistical option to address these points when comparing dynamic treatment regimens.^(14)^

Target trial emulation creates a framework for causal inference from observational data. It explicitly emulates the components of a clinical trial. It includes screening patients and identifying patients eligible for treatment allocation based on their baseline characteristics and inclusion criteria for the intervention (e.g., ICU admission). It follows patients from "randomization emulation time" (time zero) to the end of planned follow-up and conducts the same analysis as would be conducted for the corresponding target trial.^(13)^

The interventions were defined at the level of glucose trajectories rather than specific treatment actions. At each 6-hour interval, the simulated intervention enforced glucose values within the predefined target range corresponding to each strategy. This was implemented using the parametric g-formula by replacing observed glucose values with values sampled from the conditional distribution of glucose consistent with the intervention strategy and covariate history. This approach models the effect of maintaining glucose exposure within the target range, regardless of the specific clinical actions required to achieve that target, such as insulin administration or nutritional adjustments. Accordingly, the causal estimand corresponds to the per-protocol effect of maintaining glucose trajectories within the specified target range over time under full adherence, rather than the effect of specific treatment interventions used to achieve those glucose levels. The percentage of moments when an intervention was applied reflects the proportion of timepoints at which the observed glucose value fell outside the target range and was therefore replaced by a value consistent with the simulated intervention. When multiple glucose measurements were available within a 6-hour block, the mean glucose value was used to represent glycaemic exposure during that interval. The natural course represents the observed glucose trajectories and management patterns under routine clinical practice, without modification. Within the parametric g-formula framework, the natural course reflects the empirical joint distribution of glucose levels, treatments, and patient characteristics as observed in the data.

### Statistical analysis

All continuous data are reported as medians (quartile 25% - quartile 75%) and categorical data as numbers and percentages. The primary analysis was conducted using parametric g-formula. Baseline confounders included in the models were: age, sex, body mass index, admission diagnosis, presence of septic shock (according to Sepsis-3 criteria), Australian and New Zealand Risk of Death (ANZROD), usual glycaemia at hospital admission (based on HbA1c and calculated using the formula described above), total Charlson co-morbidity score, presence of diabetes, presence of chronic pulmonary disease, congestive heart failure, chronic kidney disease, highest lactate at ICU admission, use of vasopressor and/or inotropes, invasive ventilation, renal replacement therapy and Sequential Organ Failure Assessment (SOFA) score at ICU admission. Time-dependent covariates included creatinine levels, pH, mean arterial pressure, use of any oral or subcutaneous diabetic medication, use of any type of insulin and the glucose levels. All models included the hour (in blocks of 6 hours), and the time-dependent intercept was estimated by a smooth function of the day since the beginning of follow-up using natural cubic splines with five knots.

Missing baseline data from the first 24 hours after ICU admission were imputed using multiple imputation by chained equations (Table 1S - Supplementary Material). The list of predictors included the outcome variable, failure time, baseline covariates and laboratory tests. In the case of missing covariate values during the follow-up, the last observed value of a covariate was carried forward, reflecting clinical practice at the bedside where treatment decisions are based on the most recent available measurement when no new result has been obtained. In our single-centre ICU, physiological and laboratory measurements are obtained frequently, particularly during clinical deterioration, limiting prolonged reliance on carried-forward values. Subgroup analyses were performed according to diabetes (yes *versus* no), severity of illness (Acute Physiology and Chronic Health Evaluation [APACHE] III ≥ 62 *versus* < 62), type of admission (medical *versus* surgical), presence of septic shock (yes *versus* no), presence of sepsis (yes *versus* no), cardiac surgery (yes *versus* no), major surgery or trauma (yes *versus* no), and neurological admission (yes *versus* no).

As the g-formula estimates marginal risk measures, risk ratios (RR), rather than hazard ratios, are reported because the model focuses on absolute differences in risk over time rather than instantaneous rates of events. The causal contrast compares the per-protocol effect of maintaining glucose trajectories within each predefined target range with the natural course, defined as the observed glucose trajectories under routine clinical care. All analyses were performed in R Version 4.3.3 (R Foundation for Statistical Computing, Vienna, Austria).

## RESULTS

### Patients

From January 2019 until December 2023, 64,025 patients were screened for eligibility. After exclusions, 3,541 patients met the inclusion criteria and were included in the present study (Figure 1S - Supplementary Material). Most of the patients were male (64%) with a median age of 68 (59 - 76) years old and with a predicted risk of death of 5% (1% - 15%) (Table 2). Half of the patients were admitted due to surgical reasons (47%), with a median SOFA of 5 (3 - 7), 16% had sepsis, 62% received vasopressors, and 48% received mechanical ventilation. Diabetes was present in 39% of the patients and, at admission, the median HbA1c was 5.9% (5.4% - 6.6%) and the median glucose was 8.7 (7.2 - 10.6) mmol/L (Table 2). Additional baseline characteristics, including admission category and hospital source of admission are reported in table 2S (Supplementary Material).

**Table 2:** Characteristics and clinical outcomes of the included patients

			Overall (n = 3,541)
Age (years)			68.0 (59.0 - 76.0)
Male sex			2,252 (63.6)
Body mass index (kg/m^2^)			28.4 (24.8 - 33.3)
Surgical patients			1,681 (47.5)
APACHE III			55.0 (43.0 - 69.0)
ANZ risk of death (%)			4.8 (1.3 - 15.4)
Total SOFA			5.0 (3.0 - 7.0)
Sepsis*			555 (16.2)
	Septic shock*		191 (5.4)
Co-morbidities			
	Charlson Comorbidity Index		4.0 (3.0 - 6.0)
		10-year survival (%)	53.4 (2.2 - 77.5)
Diabetes			1,386 (39.1)
	Type I		116 (3.3)
	Type II		1,270 (35.9)
Chronic pulmonary disease			645 (18.2)
Chronic heart failure			636 (18.0)
Metastatic solid tumour			172 (4.9)
Severe chronic kidney disease			284 (8.0)
ICU source of admission			
	Coronary care		1 (0.0)
	Emergency department		873 (24.7)
	ICU other hospital		79 (2.2)
	ICU same hospital		13 (0.4)
	Operating room		1,662 (46.9)
	Other hospital		232 (6.6)
	Ward		681 (19.2)
Treatment limitation			454 (12.8)
Pathology tests at ICU admission			
	HbA1c (%)		5.9 (5.4 - 6.6)
	Glucose levels (mmol/L)		8.7 (7.2 - 10.6)
	Creatinine (µmol/L)		92.5 (70.6 - 138.5)
	Lactate (mmol/L)		2.2 (1.5 - 3.2)
Organ support during ICU admission			
	Vasopressors/Inotropes		2,193 (62.4)
	Invasive mechanical ventilation		1,678 (47.7)
	Renal replacement therapy		227 (6.5)
	Extracorporeal membrane oxygenation		10 (0.3)
	Trachestomy		132 (3.8)
Clinical outcomes			
Hypoglycaemia (≤ 2.2mmol/L) during ICU stay			12 (0.3)
	Episodes in all patients		0.0 (0.0 - 0.0)
	Episodes in patients with hypoglycaemia		8.0 (2.8 - 18.5)
Hyperglycaemia (> 10mmol/L) during ICU stay			1,165 (32.9)
	Episodes in all patients		0.0 (0.0 - 39.0)
	Episodes in patients with hyperglycaemia		52.0 (22.0 - 122.0)
30-day mortality			217 (6.1)
Day of mortality			3.8 (2.0 - 8.3)
Discharged from ICU alive at day 30			3,283 (92.7)
Day of ICU discharged alive			2.1 (1.0 - 4.1)
ICU length of stay (days)			2.3 (1.1 - 4.5)
In survivors			2.2 (1.0 - 4.2)
Hospital length of stay (days)			12.0 (7.1 - 21.4)
In survivors			12.3 (7.3 - 21.7)

APACHE - Acute Physiology and Chronic Health Evaluation; ANZ - Australia and New Zealand; SOFA - Sequential Organ Failure Assessment; ICU - intensive care unit; HbA1c - Hemoglobin A1c.

* Based on Sepsis-3 criteria. Data are median (quartile 25^th^ - quartile 75^th^) or n (%)

### Pathology test and medications

At ICU admission, median creatinine was 92.6 (70.6 - 138.5) µmol/L, pH was 7.38 (7.35 - 7.41), and median lactate was 2.2 (1.5 - 3.2) mmol/L (Table 3S - Supplementary Material). During ICU stay, mean, highest and lowest glucose levels were 8.7 (7.2 - 11.1), 9.5 (7.5 - 13.1), and 7.8 (6.4 - 9.4) mmol/L, respectively. The glucose levels over ICU stay are reported in figure 1.

**Figure 1:**
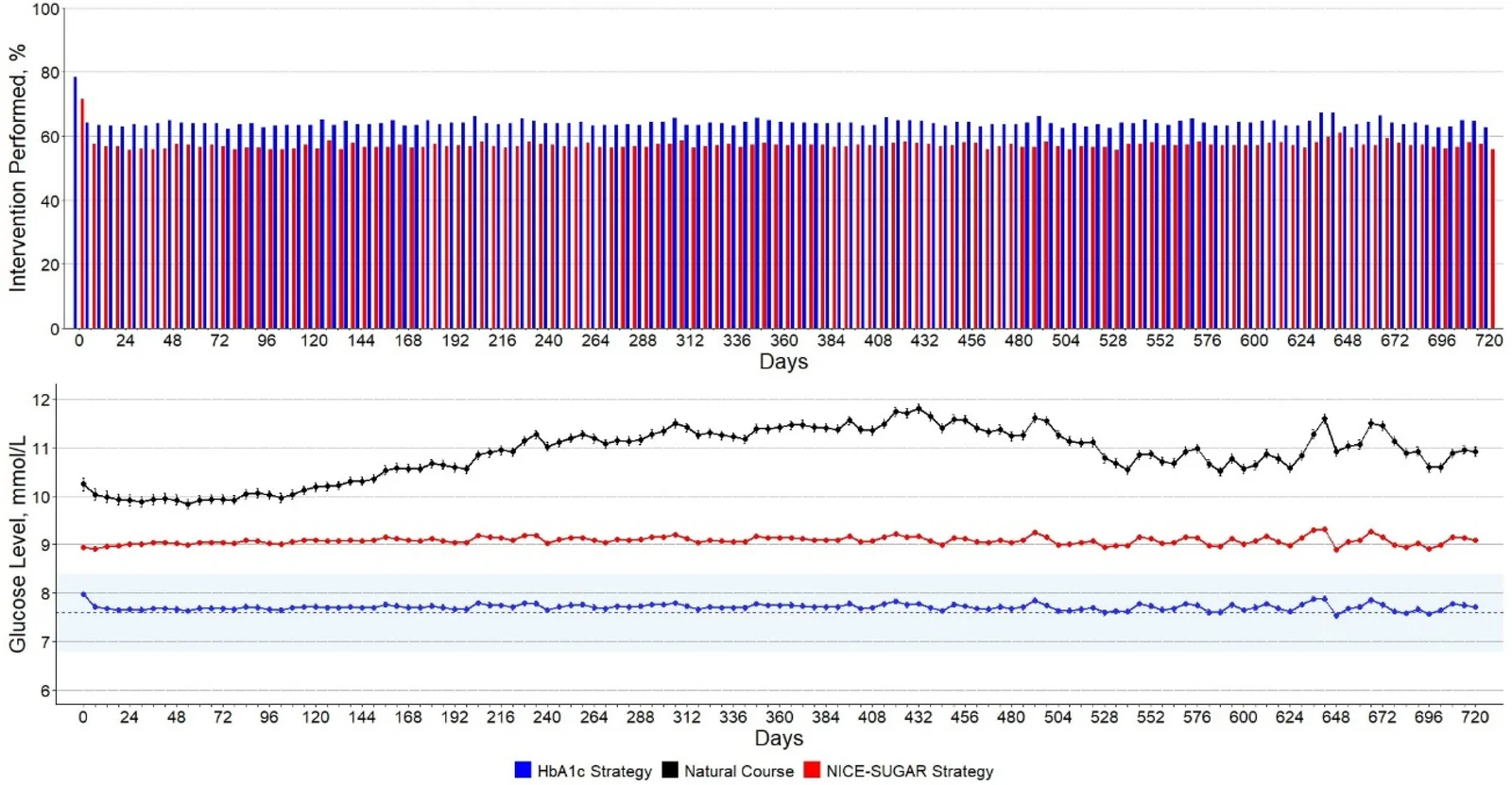
Characteristics of the interventions.

Top panel, percentage of patients where an intervention was performed at each timepoint to return the glucose levels to the proposed range in each group. An intervention was considered applied whenever the observed glucose value fell outside the predefined target range and was replaced with a value consistent with the simulated strategy. The high proportion of intervention reflects the frequency of glucose values outside narrow target ranges in routine clinical practice.

Bottom panel, median glucose levels over time stratified by treatment strategy. Blue dashed line is the mean glucose level (target) and the blue area are the thresholds for the HbA1c strategy (mean glucose ± 0.8mmol/L).

All data based on the simulated dataset computed during the parametric g-formula computation (10,000 simulated patients with data based on the 3,541 patients included in the study).

At ICU admission, 0.1% of the patients were using any non-insulin glucose-lowering medications, and 2.3% were receiving insulin, mainly rapid and short acting, and with a median dose of 8 (6 - 10) units (Table 4S - Supplementary Material). During ICU stay, 4.1% of the patients received a non-insulin glucose-lowering medications with a median time until first dose of 12 (9 - 56) hours, and 22% received insulin with a median time until first dose of 24 (12 - 57) hours, and a median dose of 8 (4 - 10) units (Table 4S - Supplementary Material).

### Clinical outcomes

Clinical outcomes are reported in table 2 and table 5S (Supplementary Material). The incidence of hypoglycaemia and hyperglycaemia during ICU stay was 0.3% and 44%, respectively. At Day 30, 6.1% of the patients had died (median time until mortality 4 [2 - 8] days) (Figure 2S - Supplementary Material) and 93% of the patients were discharged alive at a median time of 2 (1 - 4) days.

### Intervention applied on glucose levels

The simulated trajectories are presented in figure 1 and characteristics of the intervention are reported in table 6S (Supplementary Material). Blood sugar levels remained close to the proposed range in each of the defined strategies, and a good treatment separation was achieved (Figure 1 and table 6S [Supplementary Material]). The percentage of moments when an intervention was applied to correct the glucose levels was higher in the HbA1c group than in the NICE-SUGAR group (64% *versus* 57%). Mean glucose levels during treatment period in the natural course, HbA1c, and NICE-SUGAR groups were 10.9 ± 4.8mmol/L, 7.7 ± 2.7mmol/L, and 9.1 ± 0.8mmol/L, respectively. As expected, there was a higher variation of glucose levels during follow-up in the natural course than in the HbA1c, and NICE-SUGAR group (44% *versus* 35% *versus* 9%, respectively), confirming the correct application of the proposed interventions, with good adherence to the treatment (Figure 1 and table 6S [Supplementary Material]).

### Impact of blood glucose control strategy on outcome

In this target trial emulation assessing the impact of different glucose control strategies on 30-day ICU mortality, the estimated mortality under the natural course was 6.46% (95% confidence interval [95%CI] 4.16% - 8.83%). When applying the HbA1c-based strategy, mortality was 6.56% (95%CI 4.40% - 8.85%), corresponding to an absolute difference of 0.10% (95%CI -0.26% - 0.93%) and a RR of 1.02 (95%CI 0.96 - 1.16) compared to the natural course (Figure 2 and Table 7S [Supplementary Material]). Similarly, the NICE-SUGAR strategy yielded a 30-day ICU mortality of 6.52% (95%CI 4.40% - 8.82%), with an absolute difference of 0.07% (95%CI -0.15% - 0.83%) and a RR of 1.01 (95%CI 0.98 - 1.15) compared to the natural course (Figure 2 and Table 7S [Supplementary Material]). Comparable parametric *versus* nonparametric estimates of the covariate means were reported, suggesting correct model specification for the parametric g-formula (Figure 3S - Supplementary Material).

**Figure 2:**
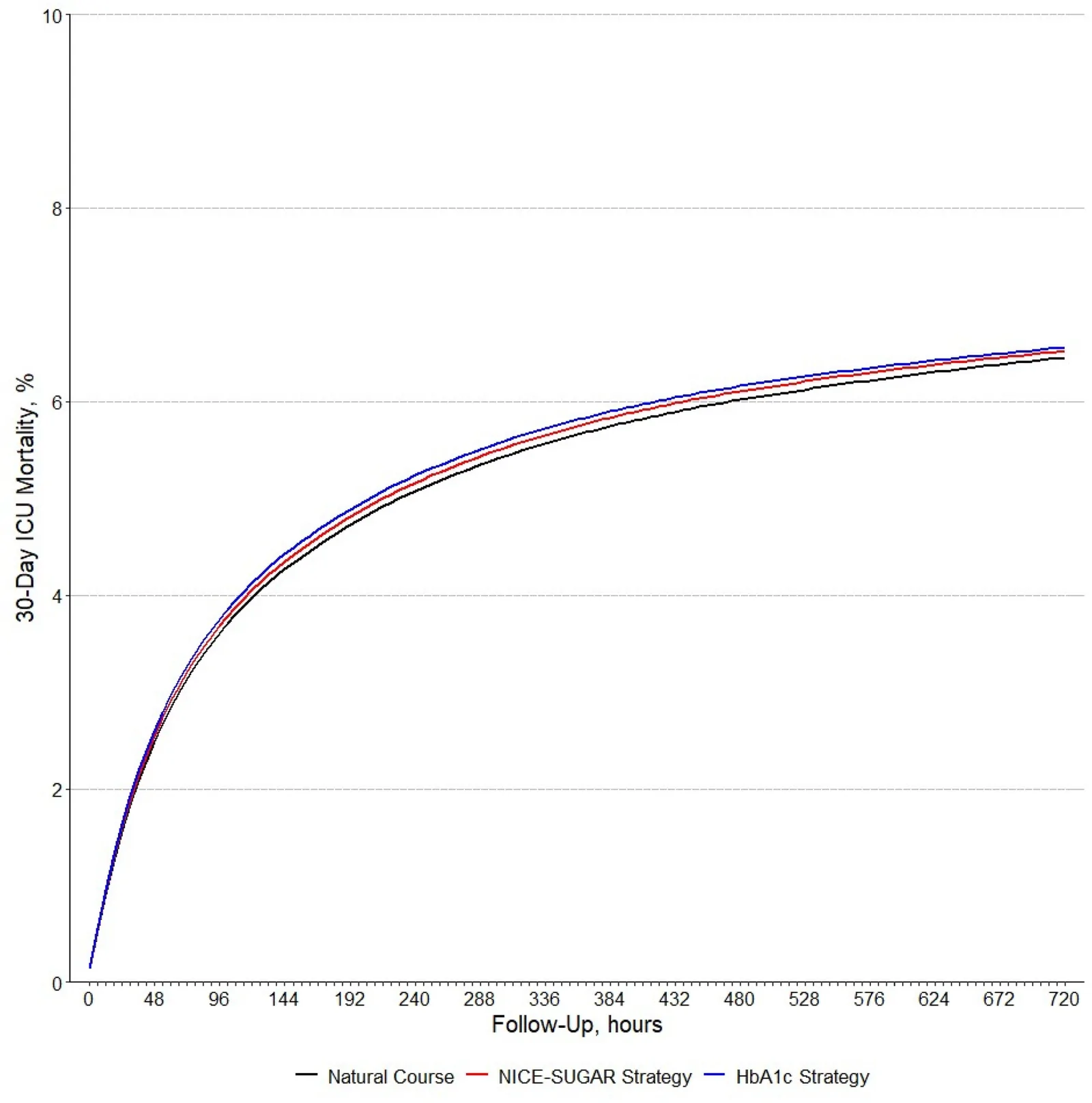
Estimated 30-day intensive care unit mortality risk under different glucose control strategies.

30-day intensive care unit mortality according to the treatment over time. Estimates from the parametric g-formula. The shaded areas represent the 95% confidence intervals. The numerical results are reported in table 2.

Subgroup analyses are reported in figure 3 and table 7S (Supplementary Material) and the findings were consistent across all subgroups, including in patients with diabetes. Because some subgroups contained relatively few mortality events, the corresponding model-based estimates show wider confidence intervals and should be interpreted cautiously. Glucose levels and simulated trajectories in patients with diabetes are presented figure 4S (Supplementary Material).

**Figure 3:**
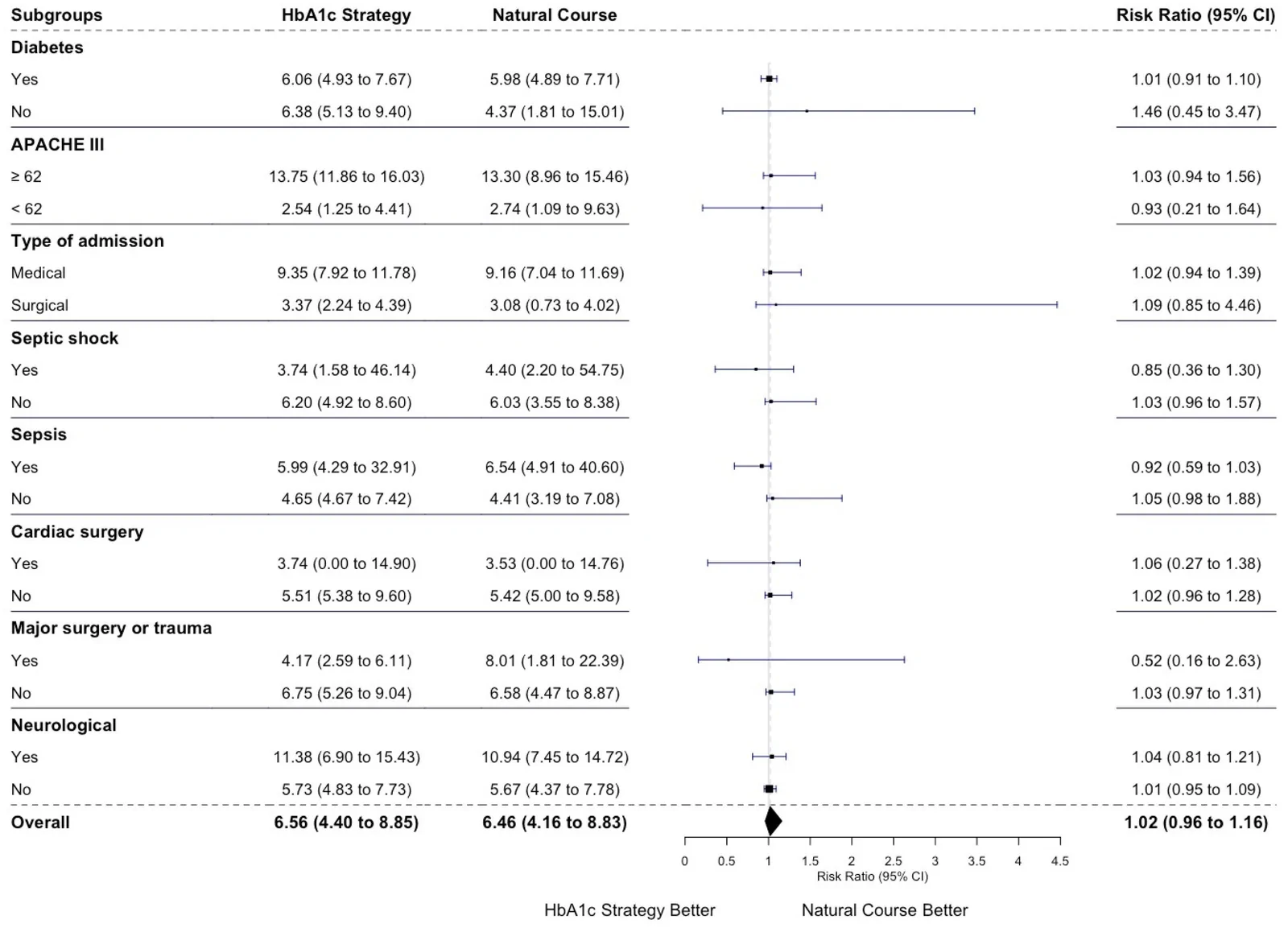
Subgroup analyses of 30-day intensive care unit mortality under different glucose control strategies.

APACHE - Acute Physiology and Chronic Health Evaluation; HbA1c - hemoglobin A1c.

Forest plot showing the impact of HbA1c strategy on 30-day intensive care unit mortality in different predefined subgroups. The findings were consistent across all the subgroups. The sizes of the data markers are proportional to the number of patients included in the analysis.

## DISCUSSION

### Key findings

In this target trial emulation using granular ICU data and the parametric g-formula, we assessed the effect of a personalized glucose management strategy, based on admission HbA1c, on 30-day ICU mortality among critically ill patients. We found no significant difference in mortality when comparing the HbA1c strategy to either usual care or the NICE-SUGAR strategy. This finding was consistent across all prespecified subgroups, including patients with and without diabetes.

### Relationship to previous studies

Our findings align with the results of the CONTROLING trial, the largest clinical trial to date examining individualized glycaemic targets in the ICU. That study also reported no survival benefit of personalizing blood glucose targets based on HbA1c.^(7)^ However, CONTROLING was limited by a delay of up to 96 hours between ICU admission and randomization, during which patients in the personalized arm received conventional glucose control. The modest separation in achieved glucose levels between study arms, and only 51% time-in-target-range in the personalized group, further diluted the potential contrast between strategies. While CONTROLING found higher mortality among non-diabetic and surgical patients in the personalized arm,^(7)^ we did not observe statistically significant subgroup differences; nonetheless, these same groups also trended toward the highest risk in our study.

Calls for high-quality research in this area are ongoing. A recent 2024 Society of Critical Care Medicine (SCCM) systematic review identified the lack of clinical trials evaluating personalized glycaemic targets as a major evidence gap.^(3)^ Prior work has highlighted the dynamic metabolic shifts in critical illness and the theoretical benefits of matching insulin therapy to an individual's baseline glycaemic physiology.^(3,15,16)^ However, translating these insights into clinical benefit has proven difficult.

Despite decades of research, the question of whether glucose control in the ICU should be individualized remains unresolved. While our study does not support mortality benefit with personalized targets, it also does not preclude benefits in other outcomes such as glycaemic variability, functional recovery, or long-term health status. The implementation of continuous glucose monitoring and automated insulin delivery systems may offer new opportunities to improve target adherence and safety, potentially realizing the vision of an ‘artificial pancreas’ in critical care. Future studies should also consider stratified enrolment by diabetic status and illness severity to better assess whether particular subgroups may derive benefit.

Although mortality was similar across strategies, the intervention strategies produced meaningful differences in glucose exposure and variability. These findings suggest that personalized glycaemic targets can alter glucose trajectories without necessarily translating into measurable survival benefits. From a clinical perspective, this supports the concept that moderate glycaemic control, as currently practiced, already achieves glucose levels within a physiologically acceptable range for most patients. More aggressive or personalized targeting may improve biochemical metrics such as glucose variability or time-in-range but may not substantially influence survival outcomes in the absence of other mechanistic benefits.

### Implications of study findings

Our findings suggest that personalizing glycaemic control based on HbA1c does not confer a mortality benefit compared to standard approaches. Both the HbA1c and NICE-SUGAR strategies yielded nearly identical outcomes to the natural course, and this lack of effect was consistent across patient subgroups, including those with diabetes. Importantly, we did not observe signals of harm with the personalized strategy, in contrast to what was reported in the CONTROLING trial, particularly among non-diabetic and surgical patients. These results indicate that while personalization may not be harmful, it is unlikely to provide major survival benefits without more advanced implementation systems.

Our findings also have important methodological and clinical implications. From a methodological standpoint, this study demonstrates the feasibility and utility of target trial emulation to explore complex, time-varying interventions in the ICU, a setting where large-scale clinical trials are often infeasible. Clinically, the results reinforce that maintaining moderate glucose control remains a safe and pragmatic approach in most ICU patients. However, as new technologies for real-time glucose monitoring and algorithm-driven insulin titration become more widely available, future investigations should revisit personalization strategies with a focus on improving time-in-target range and minimizing glycaemic variability, rather than simply achieving lower mean glucose levels.

Our cohort had a short ICU length of stay, reflecting contemporary discharge practices and case-mix at our centre. This does not imply that the g-formula extrapolated effects far beyond observed data for most patients; rather, because ICU discharge was treated as a competing event, most patients contribute limited ICU person-time during which ICU mortality can occur. Consequently, the causal contrast is concentrated in the early ICU period when glucose control is applied and when ICU deaths occur. This pattern is consistent with major glycaemic control trials, where ICU length of stay is typically only a few days,^(2,7)^ and therefore any causal effect of glycaemic strategies on ICU mortality would also be expected to emerge early rather than after prolonged ICU follow-up. Importantly, restricting analyses to patients who remain in ICU for a specific period would condition on a post-baseline variable affected by illness severity and treatment response and would not emulate a trial randomized at ICU admission, potentially introducing selection (collider) bias. We therefore focused on prespecified subgroup analyses based on baseline severity and clinical phenotypes associated with prolonged ICU stay.

### Strengths and limitations

This study has several strengths. First, we used granular, high-frequency EMR data to model dynamic glucose trajectories and apply interventions in a realistic way. Second, the application of the parametric g-formula allowed us to account for time-varying confounding and simulate interventions that mimic those of a randomized trial. Third, our cohort was large, unselected, and representative of contemporary ICU practice, improving the external validity of the findings. Fourth, our protocol incorporated rigorous treatment separation between strategies and confirmed good adherence within each simulated intervention arm, enhancing internal validity. Fifth, our consistent results across clinically relevant subgroups, including patients with and without diabetes, improve confidence in the generalisability of the primary findings. From a causal inference perspective, the parametric g-formula requires adequate overlap in covariate distributions across treatment histories. In our dataset, the observed glucose ranges and covariate distributions were well represented across exposure patterns, supporting the plausibility of the simulated interventions and reducing concerns regarding positivity violations. Finally, the inclusion of sensitivity analyses comparing parametric and non-parametric estimates provided reassurance regarding model specification.

However, several limitations must be acknowledged. The study was conducted at a single centre, potentially limiting generalisability. Our cohort was restricted to patients with an available HbA1c measurement. Although HbA1c testing at our institution is supported by an automated protocol for patients aged ≥ 54 years, younger patients or those without recent HbA1c testing may have been underrepresented. Therefore, the findings may be most generalizable to ICU populations where HbA1c testing is routinely performed or to older critically ill populations. While target trial emulation improves upon traditional observational analyses, it cannot establish causality and remains hypothesis-generating. Furthermore, although our model enforced 100% adherence to the simulated strategies, unlike real-world practice, such adherence is rarely achievable clinically. The interventions were defined at the level of glucose trajectories rather than specific treatment actions such as insulin administration. Therefore, our analysis estimates the effect of maintaining glucose exposure within predefined target ranges, rather than the effect of specific therapeutic strategies used to achieve those targets. This trajectory-based intervention reflects a conceptual causal estimand representing sustained glycaemic control under full adherence but does not capture potential risks associated with treatment actions, including hypoglycaemia resulting from insulin administration. Because glucose values were constrained within the intervention range, the risk of hypoglycaemia induced by treatment could not be directly evaluated. As a result, our findings should be interpreted as the effect of glycaemic exposure patterns rather than the safety or efficacy of specific glucose-lowering treatments. This limitation is inherent to trajectory-based causal inference methods using observational data, where interventions are defined on exposure trajectories rather than clinical actions. The glucose control observed in the natural course differed meaningfully from the NICE-SUGAR target, suggesting variability in actual practice and possible contamination of "usual care". Missing time-varying covariates during follow-up were handled using last observation carried forward. While this approach reflects clinical practice, where treatment decisions are based on the most recent available measurement, it may introduce bias if measurement frequency is related to clinical deterioration or prognosis. However, in our single-centre ICU, laboratory and physiological measurements are obtained frequently, particularly during clinical instability, reducing the likelihood of prolonged reliance on carried-forward values. Nonetheless, residual bias related to informative measurement frequency cannot be excluded. Finally, we were not able to evaluate the effect of hypoglycaemia directly, which has been a key safety concern in prior trials and may modulate any benefit of tighter control.

## CONCLUSION

In this large, single-centre target trial emulation using high-resolution intensive care unit data, we found no mortality benefit from personalizing glycaemic control based on admission HbA1c compared to standard glucose management strategies, including the NICE-SUGAR approach. The findings were consistent across all patient subgroups, including those with diabetes, and suggest that HbA1c-guided targets are unlikely to meaningfully impact 30-day resolution intensive care unit mortality under current implementation methods. While these results do not support a shift toward personalized glucose targets in routine ICU care, they highlight the importance of treatment adherence and the potential of advanced modelling techniques to evaluate complex interventions. As technologies for continuous glucose monitoring and closed-loop insulin delivery evolve, future studies should revisit the role of personalization with a focus on time-in-range, safety, and functional outcomes beyond mortality.

## Data Availability

The contents are already available
