# Supplementary material for: HbA1c-based personalization of glucose management in critical illness: a target trial emulation
Source: Crit Care Sci. 2026 Aug 31;38:e20260001. doi: 10.62675/2965-2774.20260001 (PMC13690041; doi:10.62675/2965-2774.20260001)
Supplement: Supplementary file 1 [file 2965-2774-ccsci-38-e20260001-supll01.pdf]

Critical Care Science
https://doi.org/10.62675/2965-2774.20260001

SUPPLEMENTARY MATERIAL

HbA1c-based personalization of glucose management in critical illness: a target trial emulation

Joseph Furler[1], Frank M Gao[2], Aniket Nadkarni[1], Meg Stevens[3], Dinesh Pandey[3], Hossein Jahanabadi[3], Ary Serpa Neto[1]

Table 1S - Rate of missing data

	Rate of missing data
Baseline variables	
Age	0 / 58,977 (0.0)
Sex	0 / 58,977 (0.0)
ANZROD	0 / 58,977 (0.0)
Use of vasopressors and/or inotropes	0 / 58,977 (0.0)
Invasive ventilation	0 / 58,977 (0.0)
Highest lactate at ICU admission	0 / 58,977 (0.0)
Renal replacement therapy	0 / 58,977 (0.0)
Admission diagnosis	7 / 58,977 (0.0)
Body mass index	0 / 58,977 (0.0)
Chronic pulmonary disease	0 / 58,977 (0.0)
Congestive heart failure	0 / 58,977 (0.0)
Chronic kidney disease	0 / 58,977 (0.0)
Diabetes	0 / 58,977 (0.0)
Septic shock	0 / 58,977 (0.0)
Usual glycemia at hospital admission	0 / 58,977 (0.0)
Total Charlson comorbidity score	0 / 58,977 (0.0)
SOFA at ICU admission	0 / 58,977 (0.0)
Time-dependent covariates	
Use of any type of insulin	0 / 58,977 (0.0)
Use of any oral or subcutaneous diabetic medication	0 / 58,977 (0.0)
Creatinine	40,789 / 58,977 (69.2)
Glucose	11,244 / 58,977 (19.1)
Mean arterial pressure	17,385 / 58,977 (29.5)
pH	11,244 / 58,977 (19.1)

ANZROD - Australian and New Zealand Risk of Death; ICU - intensive care unit; SOFA - Sequential Organ Failure Assessment. Data are n / total (%).

Table 2S - Additional baseline characteristics

	Overall (n = 3,541)
Additional co-morbidities	
Cerebrovascular disease	314 (8.9)
Dementia	38 (1.1)
Diabetes with chronic complications	619 (17.5)
Diabetes without chronic complications	767 (21.7)
Hemiplegia or paraplegia	156 (4.4)
Human immunodeficiency virus	3 (0.1)
Mild liver disease	354 (10.0)
Moderate or severe liver disease	161 (4.5)
Malignancy	449 (12.7)
Myocardial infarction	857 (24.2)
Peptic ulcer disease	53 (1.5)
Peripheral vascular disease	240 (6.8)
Mild or moderate chronic kidney disease	393 (11.1)
Rheumatic disease	123 (3.5)
Hospital source of admission	
Home	2720 (76.8)
Mental health	12 (0.3)
Nursing home, chronic care, or palliative care	24 (0.7)
Other acute hospital	583 (16.5)
Other acute hospital ED	74 (2.1)
Other acute hospital ICU	107 (3.0)
Rehabilitation	21 (0.6)
Broad admission category	
Cardiovascular	1163 (32.9)

Continue...

This is an open access article under the CC BY license (https://creativecommons.org/licenses/by/4.0/).

...continuation

Gastrointestinal	612 (17.3)
Gynaecological	4 (0.1)
Haematological	16 (0.5)
Metabolic	198 (5.6)
Musculoskeletal	81 (2.3)
Neurological	353 (10.0)
Renal/Genitourinary	142 (4.0)
Respiratory	515 (14.5)
Sepsis	303 (8.6)
Trauma	153 (4.3)
Specific diagnoses	
Abdominal aortic aneurysm	67 (1.9)
Cardiac arrest	97 (2.7)
Cardiac surgery	546 (15.4)
Chronic obstructive pulmonary disease	35 (1.0)
Other cardiothoracic or vascular surgeries	231 (6.5)
Gastrointestinal surgery	345 (9.7)
Neurological	180 (5.1)
Orthopedic surgery (not spine)	35 (1.0)
Orthopedic surgery (spine)	46 (1.3)
Overdose	38 (1.1)
Pneumonia	228 (6.4)
Subarachnoid haemorrhage	27 (0.8)
Seizure	51 (1.4)
Sepsis & other ICU infections (not pneumonia)	322 (9.1)
Stroke and intracranial hemorrhage	49 (1.4)
Head trauma	32 (0.9)
Non-head trauma	121 (3.4)
Other medical admissions	834 (23.6)
Other surgical admission	257 (7.3)

ED - Emergency Department; ICU - intensive care unit. Data are number (%).

Table 3S - Pathology tests

	Overall (n = 3,541)
At ICU admission	
HbA1c (%)	5.9 (5.4 - 6.6)
Glucose levels (mmol/L)	8.7 (7.2 - 10.6)
Ketones (mmol/L)	1.5 (1.0 - 2.1)
Creatinine (μmol/L)	92.5 (70.6 - 138.5)
Urea (mmol/L)	8.1 (6.2 - 12.4)
Hemoglobin (g/L)	115.2 (102.8 - 126.8)
White cell counts (x10^9/L)	10.0 (7.4 - 12.8)
Platelets (x10^9/L)	218.2 (162.1 - 294.8)
Sodium (mEq/L)	138.4 (135.4 - 139.8)
Potassium (mEq/L)	4.3 (4.0 - 4.7)
pH	7.38 (7.35 - 7.41)
Bicarbonate (mmol/L)	24.2 (22.4 - 26.0)
Lactate (mmol/L)	2.2 (1.5 - 3.2)
Mean value during ICU stay	
HbA1c (%)	5.9 (5.4 - 6.6)
Glucose levels (mmol/L)	8.7 (7.2 - 11.1)
Ketones (mmol/L)	1.3 (0.7 - 1.9)
Creatinine, μmol/L	89.0 (68.5 - 134.1)
Urea (mmol/L)	8.1 (5.9 - 12.9)
Hemoglobin (g/L)	105.9 (92.0 - 120.8)
White cell counts (x10^9/L)	10.2 (7.9 - 13.1)
Platelets (x10^9/L)	202.2 (146.9 - 268.6)
Sodium (mEq/L)	139.0 (136.6 - 140.9)
Potassium (mEq/L)	4.2 (4.0 - 4.5)
pH	7.40 (7.38 - 7.43)
Bicarbonate (mmol/L)	24.7 (22.9 - 26.5)
Worst value during ICU stay	
Highest HbA1c (%)	5.9 (5.4 - 6.6)
Glucose levels (mmol/L)	
Highest	9.5 (7.5 - 13.1)
Lowest	7.8 (6.4 - 9.4)
Highest ketones (mmol/L)	1.6 (1.0 - 2.2)
Highest creatinine, μmol/L	104.0 (78.0 - 167.0)
Highest urea (mmol/L)	10.0 (7.2 - 16.4)
Lowest hemoglobin (g/L)	95.0 (80.0 - 113.0)
Highest white cell counts (x10^9/L)	12.7 (9.7 - 17.3)
Lowest platelets (x10^9/L)	165.0 (116.0 - 225.0)
Highest sodium (mEq/L)	141.0 (139.0 - 144.0)
Highest potassium (mEq/L)	4.6 (4.3 - 5.0)
Lowest pH	7.32 (7.27 - 7.37)
Lowest bicarbonate (mmol/L)	22.4 (20.0 - 24.0)

ICU - intensive care unit; HbA1c - Hemoglobin A1c. Data are median (quartiles 25th and 75th).

Table 4S - Medications used

	Overall (n = 3,541)
At ICU admission	3,541
Any non-insulin glucose-lowering medications; n (%)	3 (0.1)
Any oral hypoglycemics	2 (0.1)
Metformin	1 (0.0)
DPP4i	1 (0.0)
SGLT2i	0 (0.0)
GLP1a	0 (0.0)
Any insulin	83 (2.3)
Intermediate	1 (1.2)
Long	7 (8.4)
Premixed	1 (1.2)
Rapid	32 (38.6)
Short	42 (50.6)
Insulin dose, U	8.0 (6.0 - 10.0)
During the first 30 days of ICU admission	
Any diabetic medication	146 (4.1)
First hour of administration	12.5 (9.0 - 56.0)
Any oral hypoglycemics	111 (3.1)
First hour of administration	13.0 (9.0 - 59.0)
Metformin	89 (2.5)
First hour of administration	18.0 (9.0 - 66.0)
DPP4i	61 (1.7)
First hour of administration	14.0 (9.0 - 45.0)
SGLT2i	6 (0.2)
First hour of administration	21.5 (10.2 - 93.5)
GLP1a	4 (0.1)
First hour of administration	34.5 (8.8 - 101.2)
Any insulin	798 (22.5)
First hour of administration	24.0 (12.0 - 57.0)
Intermediate	8 (1.0)
Long	79 (9.9)
Premixed	7 (0.9)
Rapid	416 (52.1)
Short	288 (36.1)
Mean insulin dose, U	8.0 (4.0 - 10.0)

ICU - intensive care unit; DPP4i - dipeptidyl peptidase-4 inhibitor; SGLT2i - sodium/glucose cotransporter 2 inhibitor; GLP1a - glucagon-like peptide 1 agonist. Data are median (quartile 25th - quartile 75th) or n (%)

Table 5S - 30-day intensive care unit mortality according to subgroups

	30-day ICU mortality
Diabetes	
Yes	83 / 1,386 (6.0)
No	134 / 2,155 (6.2)
APACHE III	
≥ 62	191 / 1,784 (10.7)
< 62	26 / 1,756 (1.5)
Type of admission	
Medical	167 / 1,859 (9.0)
Surgical	50 / 1,681 (3.0)
Septic shock	
Yes	17 / 191 (8.9)
No	197 / 3,320 (5.9)
Sepsis	
Yes	37 / 555 (6.7)
No	171 / 2,867 (6.0)
Cardiac surgery	
Yes	2 / 546 (0.4)
No	215 / 2,995 (7.2)
Major surgery or trauma	
Yes	18 / 576 (3.1)
No	199 / 2,964 (6.7)
Neurological	
Yes	35 / 353 (9.9)
No	182 / 3,187 (5.7)

ICU - intensive care unit; APACHE - Acute Physiology and Chronic Health Evaluation. Data are n / total (%)

Table 6S - Characteristics of the intervention*

	Natural course	HbA1c strategy	NICE-SUGAR strategy
Intervention of performed			
Percentage of moments			
Mean (95% confidence interval)	0.0 (0.0 - 0.0)	64.1 (64.1 - 64.2)	57.2 (57.1 - 57.3)
Mean ± standard deviation	0.0 ± 0.0	64.1 ± 47.9	57.2 ± 49.5
Glucose levels during follow-up (mmol/L)	10.9 ± 4.8	7.7 ± 2.7	9.1 ± 0.8
Lowest value (mmol/L)	1.1	3.0	8.0
Highest value (mmol/L)	81.0	27.7	10.0
Coefficient of variation (%)	44.4	35.1	9.2
Highest difference (mmol/L)	79.9	24.7	2.0
Amplitude (mmol/L)	70.1	20.0	0.9
Number of episodes of			
Hypoglycaemia	27,153	NA†	NA†
Hyperglycaemia	664,205	NA†	NA†

HbA1c - hemoglobin A1c; NICE-SUGAR - Normoglycemia in Intensive Care Evaluation-Survival Using Glucose Algorithm Regulation * In 10,000 simulated patients based on data from the 3,541 original patients; † number of episodes of hypo and hyperglycaemia not reported for the hemoglobin A1c and NICE-SUGAR strategy because the limits of the simulated interventions does not allow the occurrence of episodes (only in hemoglobin A1c group but as specified by the mean glucose level). Data are mean (95% confidence interval) or mean ± standard deviation.

Table 7S - Results of the target trial emulation for different glucose control strategies in the overall population and in pre-specified subgroups

		Natural course	HbA1c strategy	NICE-SUGAR strategy
All patients				
	30-day ICU mortality (%)	6.46 (4.16 - 8.83)	6.56 (4.40 - 8.85)	6.52 (4.40 - 8.82)
	Absolute difference (95%CI)	0 (Reference)	0.10 (-0.26 - 0.93)	0.07 (-0.15 - 0.83)
	Risk ratio (95%CI)	1 (Reference)	1.02 (0.96 - 1.16)	1.01 (0.98 - 1.15)
Diabetes				
Yes (n = 1,386)	30-day ICU mortality (%)	5.98 (4.89 - 7.71)	6.06 (4.93 - 7.67)	6.07 (4.93 - 7.71)
	Absolute difference (95%CI)	0 (Reference)	0.08 (-0.53 - 0.63)	0.09 (-0.52 - 0.76)
	Risk ratio (95%CI)	1 (Reference)	1.01 (0.91 - 1.10)	1.01 (0.91 - 1.12)
No (n = 2,155)	30-day ICU mortality (%)	4.37 (1.81 - 15.01)	6.38 (5.13 - 9.40)	6.30 (5.22 - 9.41)
	Absolute difference (95%CI)	0 (Reference)	2.01 (-7.78 - 5.44)	1.93 (-7.33 - 4.85)
	Risk ratio (95%CI)	1 (Reference)	1.46 (0.45 - 3.47)	1.44 (0.48 - 3.18)
APACHE III				
≥ 62 (n = 1,315)	30-day ICU mortality (%)	13.30 (8.96 - 15.46)	13.75 (11.86 - 16.03)	13.53 (11.84 - 15.70)
	Absolute difference (95%CI)	0 (Reference)	0.44 (-0.76 - 5.00)	0.23 (-0.42 - 4.55)
	Risk ratio (95%CI)	1 (Reference)	1.03 (0.94 - 1.56)	1.02 (0.97 - 1.51)
< 62 (n = 2,225)	30-day ICU mortality (%)	2.74 (1.09 - 9.63)	2.54 (1.25 - 4.41)	2.66 (1.36 - 4.64)
	Absolute difference (95%CI)	0 (Reference)	-0.20 (-7.10 - 0.90)	-0.08 (-6.78 - 0.91)
	Risk ratio (95%CI)	1 (Reference)	0.93 (0.21 - 1.64)	0.97 (0.24 - 1.67)
Type of patient				
Medical (n = 1,859)	30-day ICU mortality (%)	9.16 (7.04 - 11.69)	9.35 (7.92 - 11.78)	9.27 (7.92 - 11.75)
	Absolute difference (95%CI)	0 (Reference)	0.18 (-0.54 - 2.68)	0.11 (-0.34 - 2.56)
	Risk ratio (95%CI)	1 (Reference)	1.02 (0.94 - 1.39)	1.01 (0.97 - 1.37)

Continue...

...continuation

Surgical (n = 1,681)	30-day ICU mortality (%)	3.08 (0.73 - 4.02)	3.37 (2.24 - 4.39)	3.17 (2.25 - 4.05)
	Absolute difference (95%CI)	0 (Reference)	0.28 (-0.42 - 2.83)	0.09 (-0.21 - 2.52)
	Risk ratio (95%CI)	1 (Reference)	1.09 (0.85 - 4.46)	1.03 (0.93 - 4.03)
Septic shock				
Yes (n = 191)	30-day ICU mortality (%)	4.40 (2.20 - 54.75)	3.74 (1.58 - 46.14)	4.03 (1.98 - 46.45)
	Absolute difference (95%CI)	0 (Reference)	-0.66 (-9.02 - 1.97)	-0.37 (-5.72 - 1.52)
	Risk ratio (95%CI)	1 (Reference)	0.85 (0.36 - 1.30)	0.92 (0.61 - 1.24)
No (n = 3,320)	30-day ICU mortality (%)	6.03 (3.55 - 8.38)	6.20 (4.92 - 8.60)	6.12 (4.85 - 8.49)
	Absolute difference (95%CI)	0 (Reference)	0.17 (-0.23 - 2.42)	0.09 (-0.12 - 2.31)
	Risk ratio (95% CI)	1 (Reference)	1.03 (0.96 - 1.57)	1.01 (0.98 - 1.54)
Sepsis				
Yes (n = 653)	30-day ICU mortality (%)	6.54 (4.91 - 40.60)	5.99 (4.29 - 32.91)	6.24 (4.55 - 33.48)
	Absolute difference (95%CI)	0 (Reference)	-0.55 (-6.31 - 0.24)	-0.31 (-5.43 - 0.12)
	Risk ratio (95% CI)	1 (Reference)	0.92 (0.59 - 1.03)	0.95 (0.63 - 1.02)
No (n = 2,888)	30-day ICU mortality (%)	4.41 (3.19 - 7.08)	4.65 (4.67 - 7.42)	4.53 (4.52 - 7.16)
	Absolute difference (95%CI)	0 (Reference)	0.23 (-0.10 - 3.11)	0.11 (-0.04 - 2.82)
	Risk ratio (95%CI)	1 (Reference)	1.05 (0.98 - 1.88)	1.03 (0.99 - 1.82)
Cardiac surgery				
Yes (n = 546)	30-day ICU mortality (%)	3.53 (0.00 - 14.76)	3.74 (0.00 - 14.90)	3.47 (0.00 - 15.12)
	Absolute difference (95%CI)	0 (Reference)	0.21 (-0.73 - 1.11)	-0.06 (-2.17 - 0.61)
	Risk ratio (95%CI)	1 (Reference)	1.06 (0.27 - 1.38)	0.98 (0.30 - 1.20)
No (n = 2,995)	30-day ICU mortality (%)	5.42 (5.00 - 9.58)	5.51 (5.38 - 9.60)	5.48 (5.31 - 9.59)
	Absolute difference (95%CI)	0 (Reference)	0.09 (-0.28 - 1.56)	0.06 (-0.13 - 1.46)
	Risk ratio (95%CI)	1 (Reference)	1.02 (0.96 - 1.28)	1.01 (0.98 - 1.25)
Major surgery or trauma				
Yes (n = 729)	30-day ICU mortality (%)	8.01 (1.81 - 22.39)	4.17 (2.59 - 6.11)	4.52 (2.99 - 6.28)
	Absolute difference (95%CI)	0 (Reference)	-3.84 (-18.35 - 3.16)	-3.49 (-17.21 - 2.98)
	Risk ratio (95%CI)	1 (Reference)	0.52 (0.16 - 2.63)	0.56 (0.20 - 2.45)
No (n = 2,812)	30-day ICU mortality (%)	6.58 (4.47 - 8.87)	6.75 (5.26 - 9.04)	6.68 (5.15 - 8.96)
	Absolute difference (95%CI)	0 (Reference)	0.18 (-0.18 - 1.59)	0.10 (-0.10 - 1.51)
	Risk ratio (95%CI)	1 (Reference)	1.03 (0.97 - 1.31)	1.01 (0.99 - 1.30)
Neurological				
Yes (n = 353)	30-day ICU mortality (%)	10.94 (7.45 - 14.72)	11.38 (6.90 - 15.43)	11.21 (7.20 - 15.11)
	Absolute difference (95%CI)	0 (Reference)	0.44 (-2.34 - 2.24)	0.26 (-0.94 - 1.01)
	Risk ratio (95%CI)	1 (Reference)	1.04 (0.81 - 1.21)	1.02 (0.91 - 1.09)
No (n = 3,188)	30-day ICU mortality (%)	5.67 (4.37 - 7.78)	5.73 (4.83 - 7.73)	5.71 (4.79 - 7.76)
	Absolute difference (95%CI)	0 (Reference)	0.06 (-0.31 - 0.52)	0.04 (-0.17 - 0.39)
	Risk ratio (95%CI)	1 (Reference)	1.01 (0.95 - 1.09)	1.01 (0.97 - 1.07)

HbA1c - hemoglobin A1c; NICE-SUGAR - Normoglycemia in Intensive Care Evaluation-Survival Using Glucose Algorithm Regulation; ICU - intensive care unit; APACHE - Acute Physiology and Chronic Health Evaluation.
All models adjusted for age, sex, septic shock, the Australian and New Zealand Risk of Death (ANZROD), mean glucose level predicted by HbA1c, Charlson Comorbidity Index, presence of diabetes, chronic pulmonary disease, heart failure, chronic kidney disease, highest lactate level at admission, use of mechanical ventilation, inotropes and vasopressors, renal replacement therapy, admission diagnosis, body mass index and total Sequential Organ Failure Assessment at admission. The following time-dependent variables were included: creatinine, pH, mean arterial pressure, any oral or subcutaneous antidiabetic medication, any insulin, and glucose levels. All models included the hour in a block of 6 hours, and the time-dependent intercept was estimated by a smooth function of the day since the beginning of follow-up using natural cubic splines with five knots.

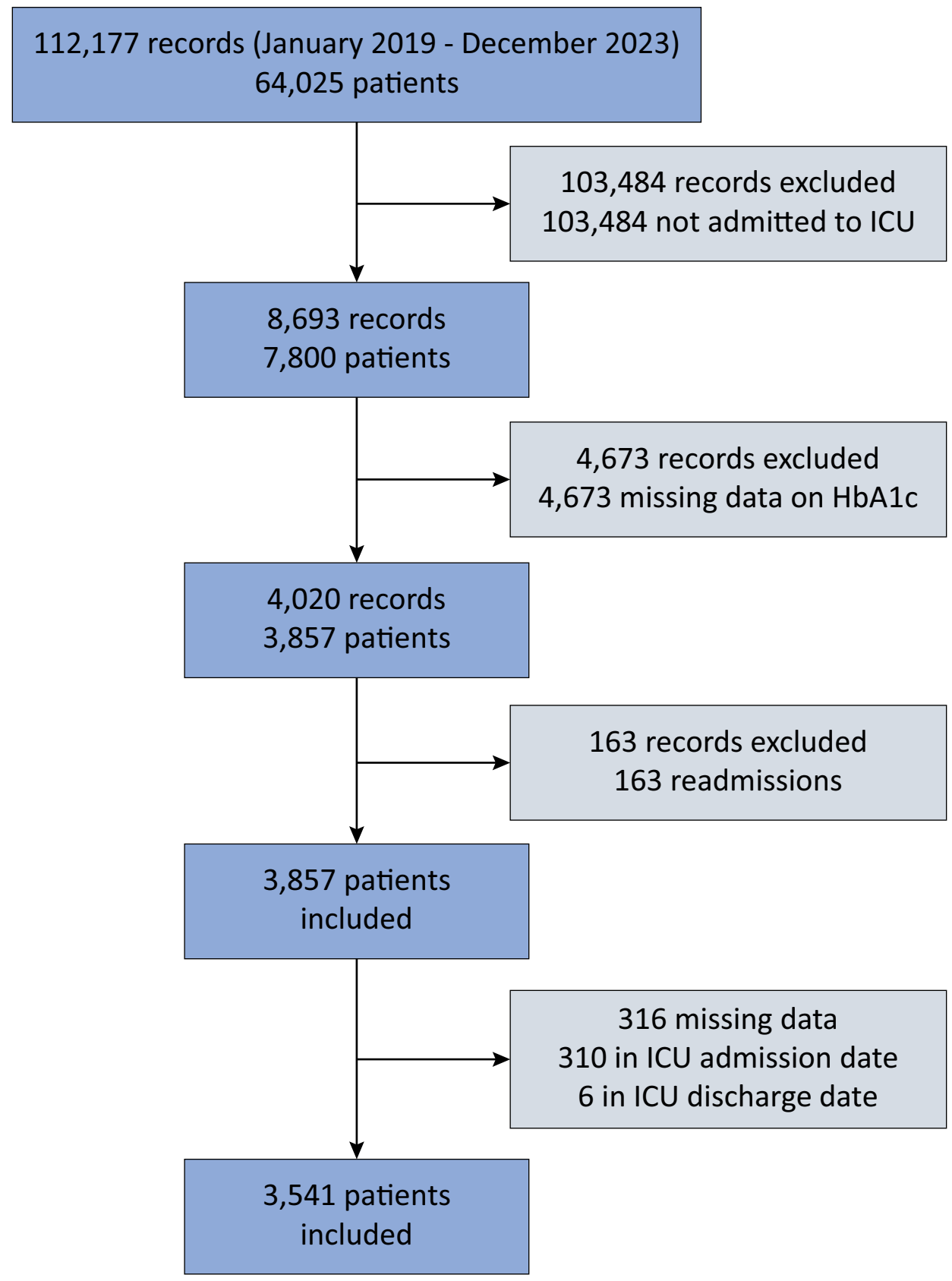

ICU - intensive care unit.

Figure 1S - Patients included in the study.

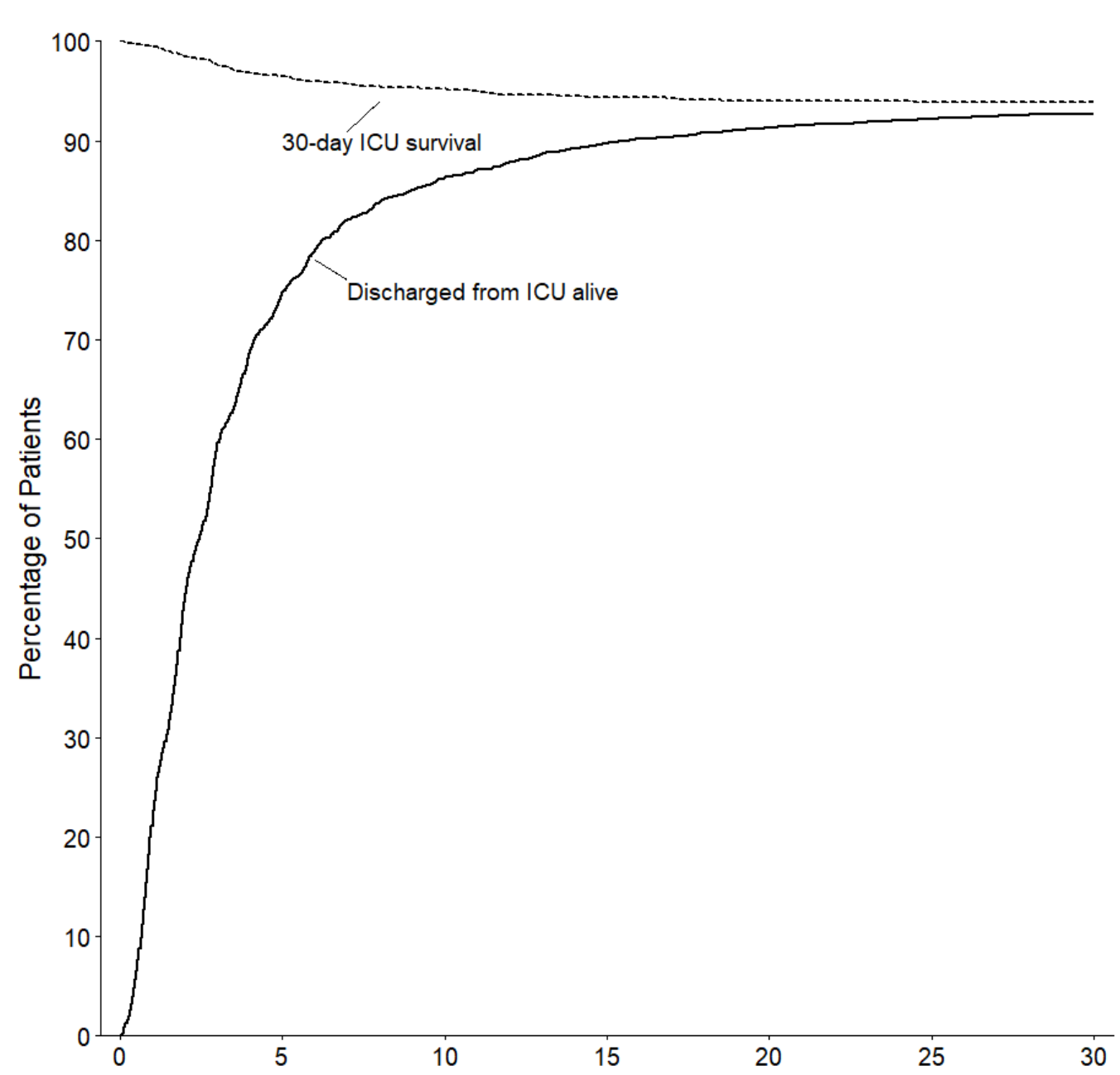

ICU - intensive care unit.

Figure 2S - Cumulative incidence plot of 30-day intensive care unit survival and 30-day intensive care unit discharge.

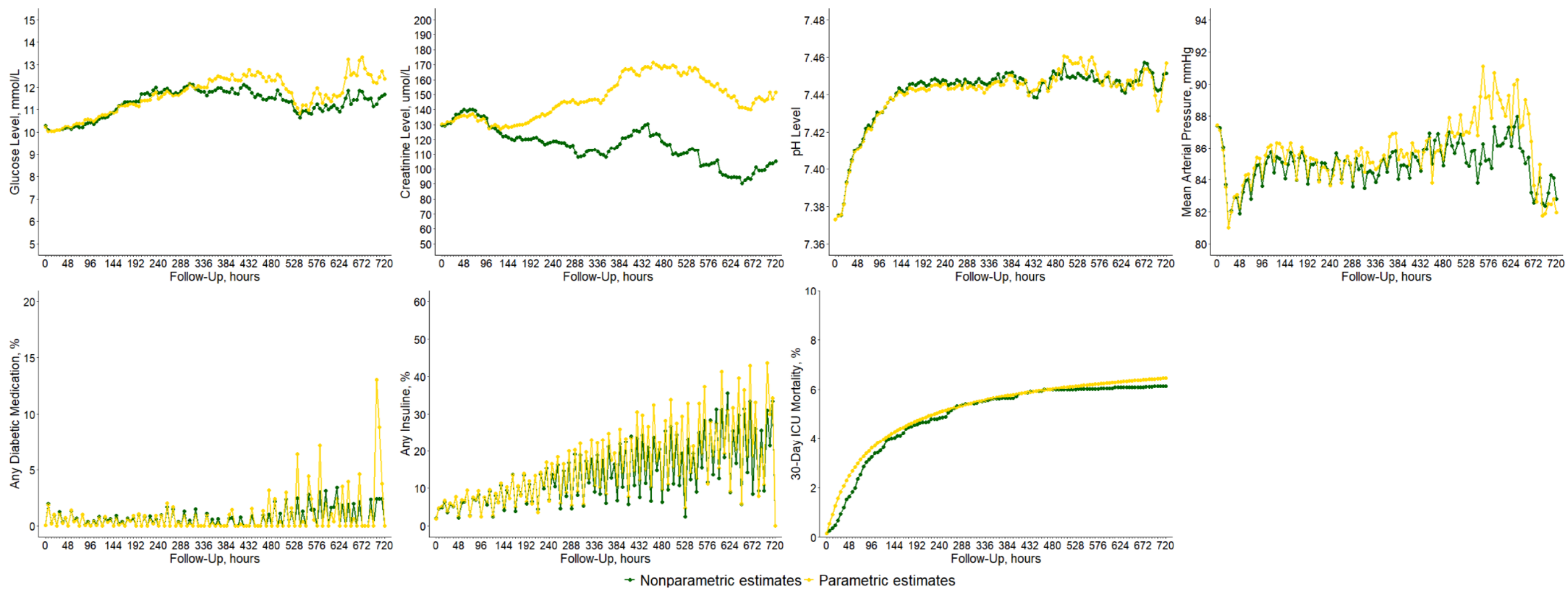

ICU - intensive care unit.

Figure 3S - Parametric *versus* nonparametric estimates of covariate means under natural course

Comparable parametric *versus* nonparametric estimates of the covariate means by each day were reported (i.e., observed *versus* predicted covariate means) for a setting without intervention (natural course), suggesting correct model specification for the parametric g-formula.

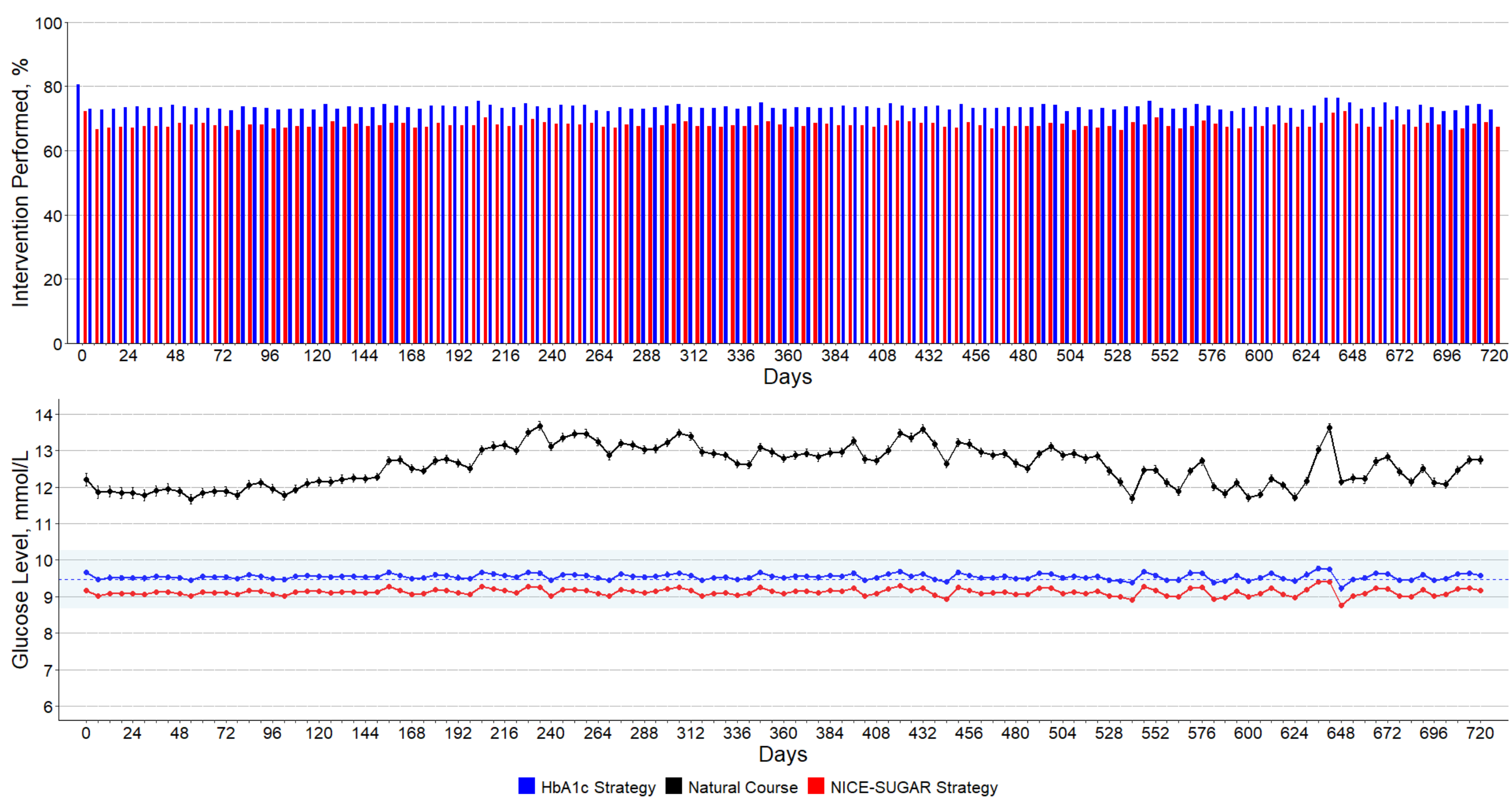

HbA1c - Hemoglobin A1c; NICE-SUGAR - Normoglycemia in Intensive Care Evaluation-Survival Using Glucose Algorithm Regulation

Figure 4S - Characteristics of the interventions in patients with diabetes.

Top panel: percentage of patients for whom an intervention was performed at each time point to return the glucose levels to the proposed range in each group.

Bottom panel: median glucose levels over time stratified by treatment strategy. The blue dashed line is the mean glucose level (target), and the blue area is the threshold for the HbA1c strategy (mean glucose ± 0.8mmol/L). All data are based on the simulated dataset generated during the parametric g-formula computation (10,000 simulated patients, based on the 3,541 patients included in the study).
